# A survey of health care needs of physicians

**DOI:** 10.1186/s12913-016-1728-4

**Published:** 2016-09-06

**Authors:** Khalid Benkhadra, Jayanth Adusumalli, Tamim Rajjo, Philp T. Hagen, Zhen Wang, M. Hassan Murad

**Affiliations:** 1 Evidence-Based Practice Research Program, Mayo Clinic, 200 First Street SW, Rochester, MN 55905 USA; 2 Mayo Clinic Robert D. and Patricia E. Kern Center for the Science of Health Care Delivery, Rochester, MN USA; 3 Division of Preventive, Occupational, and Aerospace Medicine, Rochester, MN USA

**Keywords:** Survey, Physician health, Wellness, Healthcare access

## Abstract

**Background:**

The healthcare needs of physician are not well studied.

**Methods:**

We surveyed physicians attending a large primary care conference about their access and perceived barriers to receiving healthcare services.

**Results:**

Response rate was 46 % (270/592). The majority were trained in family medicine. The age category of above 60 years was the most common (39 %) and 46 % were women. Important difficulty in accessing healthcare services was reported by 39 % of physicians and the majority (61 %) reported reverting to self-diagnosis and self-treatment. Female physicians reported more difficulties than male physicians (*p* < 0.001 for difficulty in securing access and *p* = 0.02 for self-diagnosis and treatment). The barriers cited were finding time for healthcare, concern about confidentiality, and lack of encouragement by employer. Respondents reported experiencing a career threatening illness themselves (20 %) or in a colleague (81 %). Forty-two percent experienced being concerned about a colleague being able to safely practice due to illness. Participants ranked substance abuse as the most common illnesses affecting a physician’s ability to practice followed by psychiatric disorders, heart disease, neurological disorders and cancer.

**Conclusions:**

Physicians face important barriers to accessing healthcare services. Female physicians report worse access. The identified barriers are modifiable. This survey calls for efforts to improve physicians’ health that require collaboration among physicians, employers and policymakers.

**Electronic supplementary material:**

The online version of this article (doi:10.1186/s12913-016-1728-4) contains supplementary material, which is available to authorized users.

## Background

The health and wellbeing of the practicing physician force is an important public health issue. Despite a healthier lifestyle and lower mortality than the general public reported in older studies [1], recent evidence shows that one in two physicians experience a major health issue by the age of 50 [2]. Physicians face daily challenges including irregular and long work hours [3] and are prone to stress [4], alcohol dependence, psychiatric disorders and other diseases.

Although the majority of physicians do not have financial barriers to access healthcare services, a study showed that 53 % of surveyed physicians reported having difficulty accessing health care and 63 % often revert to self-diagnosis and treatment. Over 80 % reported having or knowing a colleague who had a career-threatening illness [3]. Physicians may neglect their own health due to lack of time or concerns about confidentiality, [3] and have a significantly higher risk of dying from suicide than the general population [5]. A survey showed that although 95 % of physicians believed that working while sick put patients at risk, 83 % reported working sick [6]. Respondents would work with significant symptoms, including diarrhea, fever and acute onset of significant respiratory symptoms. The reasons cited for working sick were not wanting to let colleagues down, not wanting to let patients down, fear of ostracism by colleagues and concern about continuity of care [6].

Therefore, research on physicians’ health is important. The total population of licensed physicians in the US approaches one million [7]. Their health impacts their families’ wellbeing and their patients’ health and productivity. There is also evidence that physicians who practice healthy behaviors are more likely to counsel their patients regarding such behaviors [8]. This study aims to identify barriers that physicians report facing when accessing health care services and explore relationships between these barriers and physician demographics such as gender and working hours.

## Methods

The survey employed a handwritten questionnaire enclosed in the registration packets of attendees of a large primary care medical education and review conference in Rochester, Minnesota, USA. This conference was held in 2 sessions approximately 1 month apart with no repeat participants. Each session lasted for 3 days. The questionnaire was derived from items used in a prior survey [3] that were modified by adding additional questions. Reminder overhead announcements were made during conference breaks. Completed surveys were collected without personal identifiers via a drop box at the registration desk. No attempt was made to contact or identify any of the respondents.

Questions with response scales from 1 to 10 were summarized using means and standard deviation and were analyzed as continuous variables. Two-tailed *P* value of <0.05 was considered statistically significant. Working hours were grouped into 3 main groups: <40, 40–59 and >60 h per week and ANOVA was used to explore the relationship of working hours with other variables. T-test was used to explore the relationship between gender and other scale variables. We performed linear regression to assess the correlation between difficulty with access to healthcare and the likelihood of self-diagnosis with different barriers in getting appropriate health care. Results were reported as coefficient, 95 % confidence interval and *p* value. Results were adjusted for age, gender and number of work hours per week. Statistical analysis was done using STATA 12.1., College Station, TX: StataCorp LP.

This survey was deemed exempt by the Institutional Review Board of the Mayo Clinic (IRB application #: 15–006594). The study was funded by an intramural small grant. This was a voluntary survey and participants did not receive compensation for completion. The actual survey questions are included in Additional file 1.

## Results

The survey was distributed to 592 conference attendees. The majority were from the US Midwest. Two hundred and seventy physicians completed the survey, with an overall response rate of 46 %. Out of the physicians who completed the survey, 84 % were trained in family medicine, 9 % trained in internal medicine, and the rest were trained in surgery, psychiatry, and pediatrics. The age category of above 60 years was the most common (39 %), and about half (47 %) reported working 40–59 h weekly. Fifty-four percent were men.

### Access to healthcare services

Table 1 describes the main survey questions grouped by gender and the number of working hours per week. Around 38.9 % of physicians reported important difficulty accessing healthcare services (30.1 % of men, 49 % of women) and likelihood to revert to self-diagnosis and self-treatment (55.5 % of men, 67.7 % of women). Female physicians were more likely to report difficulty with access to health care services compared to male physicians (*p* value < 0.001), and were more likely to revert to self-diagnosis (*p* = 0.02). Physicians who worked more than 60 h a week (compared to physicians with less working hours), reported more difficulty in finding time to receive appropriate health care, more difficulty accessing health care in general, and more likely to revert to self-diagnosis .

**Table 1:** Access to care and barriers

		Gender			Working hours			
	Overall	Men	Women	*P* value	<40	40–60	>60	*P* value
Difficulty with access to healthcare^a^	3.6 ± 3	2.98 ± 2.9	4.2 ± 2.9	<0.001	2.1 ± 2.4	3.7 ± 2.9	4.5 ± 3.1	<0.001
Likelihood of self-diagnosis^b^	6.2 ± 2.8	5.9 ± 2.8	6.6 ± 2.8	0.02	5.3 ± 2.7	6.2 ± 2.8	7 ± 2.6	<0.001
Main barriers^c^								
Finding time	7.4 ± 2.6	7 ± 2.8	8 ± 2.3	0.004	6.1 ± 2.7	7.5 ± 2.6	8.4 ± 2.1	<0.001
Confidentiality	5.1 ± 3.1	4.5 ± 2.9	5.8 ± 3.2	0.002	5 ± 3	5.1 ± 3.2	5.3 ± 3.2	0.84
Inability to see someone I know	5.2 ± 3	4.7 ± 2.7	5.8 ± 3.2	0.002	5.1 ± 3	5.3 ± 2.9	5.1 ± 3.1	0.85
Not encouraged by employer	3.2 ± 2.7	2.7 ± 2.5	3.7 ± 2.9	0.006	2.6 ± 2.3	3.2 ± 2.8	3.6 ± 3	0.1

Results are expressed as mean ± standard deviation

^a^The scale used is a 10 point scale (1–10) where 1 is very easy and 10 is very difficult

^b^The scale used is a 10 point scale (1–10) where 1 is less likely and 10 is more likely

^c^The scale used is a 10 point scale (1–10) where 1 is minor barrier and 10 as major barrier

### Reported barriers

Table 2 demonstrates the association between difficulty in accessing health care and self-diagnosis, and perceived barriers. Physicians who found difficulty accessing healthcare were more likely to face barriers such as finding time (*p* value = <0.001), cost of lost work time (*p* value = 0.03) and lack of encouragement by employer (*p* value < 0.001). A similar trend was seen in other barriers but results were not significant (“confidentiality”, “seeing someone I know”, “cost of care”). Physicians who reverted to self-diagnosis were also more likely to face these barriers, with statistical significance in all the barriers except the cost of care. Female physicians ranked the following barriers to accessing appropriate health care higher than male physicians: finding time (*p* value = 0.004), confidentiality (*p* value = 0.002), seeing someone they know (*p* value = 0.002) and not being encouraged by their employer (*p* value = 0.006).

**Table 2:** Association between difficulty in accessing health care and self-diagnosis, and perceived barriers

	Difficulty with access		Likelihood of self-diagnosis	
Barrier	Coef (95 % CI)	*P* value	Coef (95 % CI)	*P* value
Finding time	0.64 (0.44, 0.84)	<0.001	0.40 (0.16, 0.63)	<0.001
Confidentiality	0.11 (−0.06 ,0.27)	0.2	0.22 (0.06, 0.38)	0.01
Inability to see someone I know	0.10 (−0.07 ,0.26)	0.25	0.19 (0.03, 0.34)	0.02
Cost of care	0.04 (−0.16 ,0.25)	0.69	0.17 (−0.03, 0.36)	0.1
Losing work time	0.18 (0.02, 0.35)	0.03	0.21 (0.06, 0.37)	0.01
Not encouraged by employer	0.37 (0.19 ,0.54)	<0.001	0.18 (0.01, 0.35)	0.03

### Experience of illness and suggested services

Twenty percent of respondents reported experiencing a career threatening illness at least once and 81 % personally knew of a colleague who had one. Around 42 % reported they have dealt with the situation of wondering whether a colleague was safe to practice medicine due to illness. Participants ranked substance abuse as the most common illnesses affecting a physician’s ability to practice. This was followed by psychiatric disorders, heart disease, neurological disorders and cancer.

In terms of the most useful services that can be implemented to improve physicians’ access to health care, participants ranked ‘rapid access for serious medical conditions’ as the most useful followed by ‘access to comprehensive care in a 1 to 2 days’.

### Free text comments

Some respondents reported that they would continue working even when they were ill because of worry about inconveniencing patients and partners. Others cited cost as a barrier because they worked in a practice that pays using relative value units system. Two respondents reported the detrimental effect on health caused by stress associated with using electronic medical records and clerical tasks. Others reported that their employer required informing administrative staff regarding personal days several months in advance. One respondent suggested providing physicians with time during the work day for exercise. Another respondent reported increased difficulty in smaller practices; particularly as it pertains to finding time and confidentiality.

## Discussion

### Main findings

The results of this study showed that physicians, despite being intimately involved in healthcare delivery, still face difficulties in accessing health care services. Barriers included finding time and to a lesser extent, concerns about confidentiality and lack of encouragement by employer. Self-diagnosis and self-treatment were a common result. Physicians who worked more hours per week reported more difficulty in finding time for their healthcare needs and were more likely to revert to self-diagnosis and treatment. Female physicians were more likely to face difficulties with access to health care, and to revert to self-diagnosis compared to male physicians. Although the cost of care is not usually thought of as barrier for a physician, some of the respondents did identify the cost of lost work time as a barrier. Substance abuse was ranked as the first illness to affect physicians’ ability to practice and many physicians have experienced career threatening illnesses in themselves or colleagues.

### Limitations and strengths

This study has several limitations. An overwhelming majority of respondents were family medicine physicians and many were older than 60 years of age; thus, results may not apply to other specialties or to younger physicians. We did not collect data on the characteristics of the physicians’ employer or practice to maintain anonymity. Open comments however suggested that physicians working in smaller and isolated practices had greater difficulty in finding time to seek healthcare services and concerns regarding confidentiality. The strengths of this study include a reasonable sample size. We almost doubled the size of a similar survey [3] and addressed some of its limitations by adding questions to allow stratification by age, gender and work hours. Although a response rate of 46 % is generally acceptable in surveys similar to this one [9, 10], we have no data on nonrespondents or physicians that did not attend this educational conference. Nonrespondents may have different perceptions or barriers to healthcare. Nonetheless, recent empirical findings illustrate cases in which the linkage between nonresponse rates and nonresponse bias is absent [11]. Therefore, we don’t believe that inferences from this survey are highly affected by nonresponse bias.

### Practical implications

This survey shows that physicians face important barriers to healthcare services. The situation has not improved and the barriers remain unchanged when compared to the survey conducted a few years ago [3]. For practical purposes and from a quality improvement perspective, all the identified barriers are modifiable. Lack of time to pursue healthcare services, worry about confidentiality and the desire to see a physician of choice, are all factors that can be accommodated by employers. Collaboration among physicians, employers and policymakers will be needed however to make meaningful changes.

Dealing with physicians as an occupational group or a population with special health needs can be done in the context of an employee health clinic or occupational health practice. Such clinics can develop expertise in providing physicians with interventions to reduce burnout and improve health, such as mindfulness and resiliency interventions, exercise programs and preventive services [12–14]. Participants in this survey have identified some of these strategies on their own and brought up in their narrative comments exercise, scheduling changes to allow for medical visits, and the need to reduce stress associated with using electronic medical records and clerical tasks.

Issues pertaining to the health of physicians have been under increasing scrutiny in the past few years. Physicians with healthy behaviors are more likely to counsel their patients about preventive measures and healthy life style. A study has shown that patients found physicians who disclosed healthy personal habits to be more motivating and believable [15]. It is plausible to expect a similar motivation in patients of physicians who disclose good preventive behaviors with regards to regular clinic visits and appropriate preventive screening.

Our finding of particularly poor access to healthcare reported by female physicians is troubling. The Accreditation Council for Graduate Medical Education (ACGME) reports an increase in female residents from 35 % in 2001–02 to 42 % in 2009–10; and the federation of State Medical Boards (FSMB) shows that at least 32 % of physicians with an active license in 2014 are women [7]. One study found that female surgeons have a higher point prevalence of alcohol abuse than their male counterparts [16]. Unfortunately such disparities are documented for non-physician women as well [17].

## Conclusions

Physicians face significant barriers to accessing healthcare services. Female physicians report more difficulty with such access. The identified barriers are modifiable. This survey calls for efforts to improve physicians’ health that require collaboration among physicians, employers and policymakers. Survey participants and the literature suggest various strategies including interventions to reduce burnout and improve health, such as mindfulness and resiliency interventions, exercise programs and preventive services, scheduling changes to allow for medical visits, and the need to reduce stress associated with using electronic medical records and clerical tasks.

## Additional file

Additional file 1: Survey/questionnaire used in this study. File contains the actual survey questions. (DOCX 18 kb)

## Abbreviations

ACGME: Accreditation Council for Graduate Medical Education
Coef: Coefficient
FSMB: Federation of State Medical Boards
