# Supplementary material for: A survey of health care needs of physicians
Source: BMC Health Serv Res. 2016 Sep 6;16(1):472. doi: 10.1186/s12913-016-1728-4 (PMC5013614; doi:10.1186/s12913-016-1728-4)
Supplement: Additional file 1: — Survey/questionnaire used in this study. File contains the actual survey questions. (DOCX 18 kb) [file 12913_2016_1728_MOESM1_ESM.docx]

**Additional file 1**

***Survey Questions***

Please mark your answer by circling the number directly on the scale from 1-10

**1. How well do physicians in general prioritize care of their own health?**

Very Low Priority 1-2-3-4-5-6-7-8-9-10 Very High Priority

**2. How well do you prioritize caring for your own health?**

Very Low Priority 1-2-3-4-5-6-7-8-9-10 Very High Priority

**3. How difficult is it for you to find time and get access to appropriate health care?**

Very difficult 1-2-3-4-5-6-7-8-9-10 Very Easy

**4. How often do you or colleague revert to self-diagnosis /self-treatment because of barriers in getting care?**

Never 1-2-3-4-5-6-7-8-9-10 Very Often

**5. What do you see as the main barriers to physicians getting appropriate health care?**

Finding time Minor Barrier 1-2-3-4-5-6-7-8-9-10 Major Barrier

Confidentiality Minor Barrier 1-2-3-4-5-6-7-8-9-10 Major Barrier

Seeing someone I know Minor Barrier 1-2-3-4-5-6-7-8-9-10 Major Barrier

Cost of care Minor Barrier 1-2-3-4-5-6-7-8-9-10 Major Barrier

Cost of lost work time Minor Barrier 1-2-3-4-5-6-7-8-9-10 Major Barrier

Not encouraged by employer Minor Barrier 1-2-3-4-5-6-7-8-9-10 Major Barrier

Don’t believe in it Minor Barrier 1-2-3-4-5-6-7-8-9-10 Major Barrier

Other_____________ Minor Barrier 1-2-3-4-5-6-7-8-9-10 Major Barrier

**6. Have you ever had a career threatening illness?** ____YES ____NO

**7. Have colleagues you know had a career threatening illness?** ____YES ____NO

**8. Have you dealt with the situation of wondering whether a colleague is safe to practice?**

____YES ____NO

**9. Have you or your practice ever needed referral to assess an ill colleague?** ____YES ____NO

**10. Rank the following illnesses in order of how commonly you think they affect a physician’s ability to practice? (1=most common, 2=second most common …….)**

___Psychiatric Illness ___Substance abuse ___Cancer ___Heart disease

____Neurological disease/Cognitive problems ___ Other_____________________

**11. How would you rate the usefulness of the following services to you?**

Rapid/Walk in access to Preventive Care. Not at all Useful 1-2-3-4-5-6-7-8-9-10 Very Useful

Access to comprehensive care in a 1-2 day visit Not at all Useful 1-2-3-4-5-6-7-8-9-10 Very Useful

Confidential Phone consult regarding health concerns Not at all Useful 1-2-3-4-5-6-7-8-9-10 Very Useful

Rapid referral access for yourself or a colleague with a serious medical condition

Not at all Useful 1-2-3-4-5-6-7-8-9-10 Very Useful

Access to care during or adjacent to a CME course Not at all Useful 1-2-3-4-5-6-7-8-9-10 Very Useful

Advising, counselling, training services related to Career and Work / Life balance

Not at all Useful 1-2-3-4-5-6-7-8-9-10 Very Useful

A Specialized Clinic dedicated to the health needs of physicians

Not at all Useful 1-2-3-4-5-6-7-8-9-10 Very Useful

A Research Program dedicated to the health needs of physicians.

Not at all Useful 1-2-3-4-5-6-7-8-9-10 Very Useful

**12. Your Age (circle age group) -** 25-30 31-35 36-40 41-45 46-50 51-55 56-60 61-65 >65

**13. Your Gender** ______Male ______Female

**14. Your speciality?** ______________________________

**15. How many hours per week do you work on an average:**

<40 40-59 60-79 80-99 100-109 110 and above

**16. How many Children/dependents do you primarily care for? ___________________________**

**17. Other suggestions or thoughts (please continue on the back of this sheet)**

**------------------------------------------------------------------------------------------------------------------------------------------**

**------------------------------------------------------------------------------------------------------------------------------------------**

**------------------------------------------------------------------------------------------------------------------------------------------**

**------------------------------------------------------------------------------------------------------------------------------------------**

**------------------------------------------------------------------------------------------------------------------------------------------**

**------------------------------------------------------------------------------------------------------------------------------------------**

**------------------------------------------------------------------------------------------------------------------------------------------**

**------------------------------------------------------------------------------------------------------------------------------------------**

**------------------------------------------------------------------------------------------------------------------------------------------**

**------------------------------------------------------------------------------------------------------------------------------------------**

**------------------------------------------------------------------------------------------------------------------------------------------**

**------------------------------------------------------------------------------------------------------------------------------------------**

**------------------------------------------------------------------------------------------------------------------------------------------**

**------------------------------------------------------------------------------------------------------------------------------------------**

**------------------------------------------------------------------------------------------------------------------------------------------**

**------------------------------------------------------------------------------------------------------------------------------------------**

**------------------------------------------------------------------------------------------------------------------------------------------**

**------------------------------------------------------------------------------------------------------------------------------------------**

**------------------------------------------------------------------------------------------------------------------------------------------**

**------------------------------------------------------------------------------------------------------------------------------------------**

**------------------------------------------------------------------------------------------------------------------------------------------**

**------------------------------------------------------------------------------------------------------------------------------------------**
